# Hospital and Institutional News

**Published:** 1915-10-09

**Authors:** 


### HOSPITAL AND INSTITUTIONAL NEWS.

### THE POOR BABIES OF LAMBETH.

The Local Government Board have sanctioned a proposal of the Lambeth Board of Guardians that Miss A. N. V. Johnson, medical officer at their Norwood schools, should receive an additional £25 per year for her services as medical officer to their nursery recently inaugurated at Norwood. This new departure is due to the insistence of the Local Government Board that all babies should be removed to Norwood from the workhouses in North Lambeth. Some eighty babies have been transferred, and they demand much care and attention, both medical and nursing. Dr. Johnson's long experience with the children at the schools naturally qualifies her for the post of medical attendant to these babies, who cannot fail to be benefited by her care. The extra remuneration of £25 is not in any sense an adequate compensation for the medical officer.

### DECREASE IN INFIRMARY PATIENTS.

The Birmingham Guardians are not alone in their experience of a decrease in the number of patients in their infirmaries. In Birmingham investigations lead the Guardians to believe that the large reduction may be due to three causes. In the first place, men with slight ailments are not now going into infirmaries, as on account of the demand for labour and the high wages paid they are continuing to work; second, by reason of there being more money in different households men and women are doctoring themselves for slight ailments; and, in the third place, the probability that the additional money is being used for buying more food is a factor that must not be overlooked. No doubt these three causes are at work in other industrial districts, and it is satisfactory to know that at this time of shortage of doctors and nurses there is also a shortage of infirmary patients.

### TO HELP THE BRITISH WOUNDED.

Plans for the celebration of October 21 as a day for assisting the work of the British Red Cross Society and the Order of St. John in helping the wounded on the battlefields of Flanders, France, and Gallipoli are well laid. In practically every town and village in the United Kingdom there is a band of workers, but anyone willing to do something for a disabled soldier should write to Miss May Beeman, the organising secretary of the movement, 10 West Bolton Gardens, Earl's Court, London, indicating the services he or she is prepared to render.

### A NEW SUPERINTENDENT AT THE PARK HOSPITAL.

To the Park Hospital, Hither Green, one of the fever hospitals of the Metropolitan Asylums Board, the appointment of a new medical superintendent is announced. Mr. Clifford Price, M.R.C.S,, L.R.C.P., has been appointed to the post left vacant by the retirement of Dr. Birdwood last year. Mr. Price, who has been an assistant medical officer under the Board for some years, has been acting-superintendent at the Park Hospital since last autumn, when the institution reverted to the fever service from being a hospital for sick children. Mr. Price qualified in 1901, was for some time attached to the North-Eastern Hospital, and has had considerable experience of fever work. His promotion to the post of medical superintendent is an instance of an unusually rapid rise in the service of the Metropolitan Asylums Board, though in view of the fact that he has already been acting-superintendent for ten months or so his appointment is not a matter for much surprise.

### MANCHESTER'S BURDEN.

The shortage of medical practitioners has led to a very unfortunate result in Manchester, for we learn that the Parker Street out-patients' department is to be closed for that reason on October 11. It is to be noted that the out-patient department at the Royal Infirmary itself is not to be closed, but only the branch establishment. A very high percentage of the medical staff have left the infirmary in order to give their services to the Army, and the whole of the honorary staff, with one exception, are engaged on military work, though in some cases it is not whole-time work. The infirmary authorities are much exercised at the appeals of the War Emergency Committee for more medical officers. The fact is, as we noted last week, that the Army authorities could and must find a way to greater economy in their disposition of doctors; and the same consideration applies to the voluntary hospitals, including the Manchester Infirmary. But surely Sir William Cobbett was misreported as saying that if the Manchester Infirmary were deprived of any more doctors the "difficulties of carrying on the war would be insuperable."

### NEW ZEALAND PRACTITIONERS AND MILITARY SERVICE.

The medical profession in New Zealand is dissatisfied with what appears to be the imperfect method employed in that Dominion for organising the profession for war service. The director of military hospitals reported to the Government that he was prepared, if necessary, to organise and nationalise the profession so that medical men might be moved from a district comparatively well supplied with doctors to another district less happily situated in this respect. For some reason or other medical men were not consulted before the report was presented, and the profession is very properly annoyed at this oversight. The Council of the New Zealand Association has, however, formulated certain proposals which will meet with the approval of the profession. According to the *New Zealand Medical Journal*, the present number of doctors is no more than barely sufficient to supply the needs of the Dominion itself and of its forces overseas. The Association, however, is not in a position to provide medical men for service in the United Kingdom.

### WINCHMORE HILL SANATORIUM CHANGES.

Chiefly for financial reasons the London Insurance Committee have surrendered twenty-five of the beds reserved for insured female consumptives at the Winchmore Hill Sanatorium. That the funds of this committee are in an unsatisfactory state has been recently announced in the newspapers, and it was only to be expected that expenditure would be cut down. Nevertheless it may reasonably be supposed that during the last three years some impression has been made on the arrears of work, and that now there is not so long a waiting list of insured tuberculous persons as was formerly the case. There has been no change made in the number of beds in use for tuberculosis at Winchmore Hill, for those beds liberated as mentioned above have been placed at the disposal of the London County Council for uninsured tuberculous patients. At this hospital there are also a certain number of beds reserved for consumptives sent by the Middlesex County Council, accommodation being afforded for both male cases and female cases. This authority is, we believe, desirous of obtaining for its cases a considerably increased number of beds at Winchmore Hill.

### AUSTRIA'S ARMY OF CRIPPLED SOLDIERS.

Of the terrible havoc wrought by the war among the male populations of Germany and Austria we occasionally get glimpses through the medical Press of neutral countries. Thus one reads in a letter from Vienna of large armies of crippled soldiers, and of the movement that has sprung up in the central European countries to supply these victims of Teutonic madness with substitutes for their lost limbs. In Austria two of Vienna's most distinguished surgeons are at the head of the movement. They first of all collected a large percentage of the cases needing artificial limbs, and a regular limb factory has been established in one of the surgical clinics, where upwards of forty skilled men are busily engaged in the production of these appliances. The soldiers are taught how to use their new limbs, and in order to show what it is possible for a limbless man to achieve, regular demonstrations are given by a man who lost both arms and feet in an accident, and now by means of four artificial limbs is able to do all the work he used to do before the mishap; and he also teaches the soldiers, and encourages them to practice. Already there are to be seen in Austrian fields labourers who, it is said, do not work less than their normal brethren, working with tools held in artificial hands, and regarding their stumps of legs as minor troubles.

### A CHARMING FIRST NUMBER.

One of the most charming "first numbers" we have seen for many a day is the *Gazette* of the 3rd London General Hospital, Wandsworth, a copy of which has reached us this week. From beginning to end it is full of bright interest, and its general appearance and arrangement suggest that among the staff is someone who wields the pen in time of peace. An excellent illustration of the catholicity of our New Army is to be found within the pages of this little magazine, whose articles appear to be written by men of widely divergent classes, whose pictures are worthy of some of our best journals, and whose verses, in some instances, possess abundant merit. The lighter and humorous side predominates, and this is as it should be; for laughter is better medicine than tears for our sick and wounded heroes. But although jokes—and really good ones, too—occupy much space, the serious side of hospital life is not overlooked. Thus the commanding officer, Lieut.-Colonel Bruce Porter, tells how the hospital came into being, and the matron, Miss Barton, relates her experiences of her "First Day at the 3rd," while another writer gives his impressions of the visit paid to the hospital by their Majesties the King and Queen. But space will not permit us to deal as fully with our new contemporary as we would wish, so we will conclude with heartiest congratulations to all concerned in its production.

### HOSPITAL FARMING IN LONDON.

We have always maintained that the basis of voluntary hospital finance must be the continued interest of the people in the district in their institution. From this obvious principle follows the need of making the hospital a household word in the locality, in the sense that every member of the public is personally conversant with the details of its work, and thereby appreciates, from a personal point of view, the difficulties to be faced, and the actual needs which lead to a public appeal for money. A hospital, then, must court publicity not so much by advertisement, as usually understood, but by interesting everyone in its doings. This policy, rightly understood, makes the organisation of its appeals a process by which the public even more fully is led to take an inside view of its affairs. One of the London institutions which has generally acted on this principle is the Royal Hospital for Incurables, Putney Heath, and the appeals annually issued from it at this time of year form a series which show how much interest can be aroused by making the inner life of the institution a matter of public knowledge. This year, for instance, there has reached us a carefully drafted pamphlet, rich in illustrations, called "A Hospital Farm in London." Anyone would read it for its interest, and very few, we believe, will lay it down without realising in fuller measure the many-sidedness of the hospital's work, from which an easy corollary is the importance of maintaining it in full activity.

### ABLE APPEALS DRAFTING.

Considered technically as an able piece of appeal drafting, the pamphlet, "A Hospital Farm in London," is mainly of interest as showing that its object—that of attracting support to the hospital—is not thrust forward, but allowed to emerge as an inevitable deduction from the interest of the story it presents. The details, showing how much produce can be home-grown in a hospital garden with any ground to spare, are particularly arresting to-day when the need for increased production at home is apparent to every householder, and when, for instance, almost everyone outside the cities is asking himself whether it is not possible for him at least to keep fowls! The illustrations are cleverly chosen, and the portrait of Mr. John Thatcher, who has worked on the hospital's twenty-five acres for almost fifty years, is not only, we fancy, a good portrait, but also one which strikes the right note at the outset. Mr. Thatcher suggests not only a farm but a prosperous one, and indeed we learn that by the sale of pigs and hay, and the supply of vegetables to the hospital ("charged" at current market prices) last year a profit of £84 was made. The haystack, the fowls' run, the vinery, the plant "ward," the orchard, the animals, the account of the finding of a pheasant's nest in the grounds form the details out of which the story of "A Hospital Farm in London" is built. All hospitals do not possess farms, but every hospital possesses material round which more than one informing pamphlet can be written concerning this or that aspect of its work. Some years ago we remember that the same institution issued a series of "Thoughts" to which its patients had contributed, and by which an insight into their active mental lives was provided. These two widely different subjects, both arising out of the daily inner work of the hospital, show how rich in interesting material every hospital really is. To arrange this for publication is the way to interest the public. A capital illustration of how this may be done is provided in this excellent pamphlet in which the word "appeal" is so neatly tucked away.

### HOSPITALS AND THE SPIRIT TAX.

As will be seen from our Parliamentary news elsewhere in this issue, the Chancellor of the Exchequer has notified his intention to bring before the House of Commons proposals with reference to a "grant" to hospitals in respect of the spirit duty. Whether or not this intention will be carried out at an early date is not stated, and we should have been better pleased if Mr. McKenna had added that there would be no delay in bringing this matter forward. In the meantime we notice that there is published in the Supplement to the current issue of the official organ of the British Medical Association—the organisation which was mainly responsible for the withdrawal of the Government's original proposal—the draft of a new sub-clause which is said to meet with the approval of the medical secretary of the Association, the secretary of the Pharmaceutical Society, as well as Mr. Bridgeman. The first paragraph of this draft is as follows:—"If the treasurer or other responsible officer of a public hospital shows to the satisfaction of the Treasury that any tinctures or other articles which contain spirits or in the preparation or manufacture of which spirits are used have within the twelve months preceding the thirty-first day of July nineteen hundred and fifteen been consumed for medical purposes in the hospital, the Treasury may, subject to such conditions and regulations as they may prescribe, pay to the treasurer or officer out of moneys provided by Parliament an allowance equal to the amount which is shown to their satisfaction to have been paid by way of duty in respect of the spirits contained in or used in the preparation or manufacture of those articles." Now let us compare these proposals with those embodied in the clause which the Government desired to insert in a Bill nearly three months ago, and which was printed in The Hospital of June 26 (page 269). The main difference appears to be that, whereas the Government intended to repay to hospital authorities half-yearly the amount the hospital had paid in respect of the duty on spirit actually consumed in the preceding six months, it is proposed by the new clause to take as the permanent basis of the repayment the quantity of spirit used during the year ended July 31, 1915. Readers will no doubt remember that in a letter which appeared in The Hospital of July 24 (page 359), Mr. N. Bishop Harman, chairman of the Parliamentary Sub-Committee of the British Medical Association, said that one of the grounds for the Association's opposition was that it was bad policy "to base a grant to a hospital on the amount of alcohol which it could contrive to consume." We pointed out at the time that the use of the word "contrive" in this connection constituted a slander on the voluntary hospital system; but apparently the Association is unrepentant, since the clause it has agreed to has been deliberately worded so as to prevent any encouragement to hospitals to waste spirit.

### THE B.M.A. AND THE VOLUNTARY HOSPITALS.

Seeing that there was never any real ground for supposing that hospitals would be extravagant in the use of spirit if it were freed from duty, we fail to see that the Association was justified in jeopardising the concession offered by the Government. It is, of course, obvious that the Association has done its best to "climb down" gracefully, and when we consider that the amendment now proposed by the Association might have been made in the original clause more than two months ago, we can only regard the attitude of the Association throughout as unwarranted. No doubt it was based upon ignorance and perversity rather than upon any real intention to deprive the hospitals of a most valuable legacy. The word "grant" has been freely used in connection with this proposed repayment of duty to hospitals. Possibly there is some subtle object in this, and we note that the proposed new clause provides that the repayment is to be made by "the Treasury," and not by the "Commissioners of Customs and Excise." Whether all this talk of a grant to hospitals is leading up to the question of the State subsidy of hospitals we do not know, but it is quite obvious that if the Treasury merely hands back to hospitals moneys that the hospitals have paid to the Treasury, there can be no question of a grant or of a subsidy, and that the use of the word "grant" in this connection is wrong. For the rest we note that the definition sub-clause has been revised so as to preclude the possibility of any institution carried on for the purposes of gain taking advantage of this concession. This, again, was an amendment which could have been introduced two or three months ago. Throughout the whole of this unfortunate business the action of the British Medical Association has been unaccountable.

### "AN UNFAIR BURDEN."

With reference to the Note appearing under this heading in our issue of last week (page 7), a Liverpool correspondent suggests that we fell into an error. He writes: "You speak of the numbers of young men who have joined His Majesty's Forces, and continue, 'Now that they have gone their contributions cease.' Surely this is not so; at any rate, as regards the troops quartered in this country, from whose modest pay regular deductions continue to be made on account of National Health Insurance, although it is extremely difficult to see where the corresponding benefit comes in." Our correspondent has misread our Note, and missed its point. It is perfectly true that the payment of contributions to Insurance Committees for Medical Benefit in respect of soldiers has ceased. Medical provision is made for the Forces from Army funds, and consequently there is no need for the provision from National Health Insurance funds, and as a corollary no contributions for medical benefit are exacted from soldiers. At the same time we would remind our correspondent that soldiers are still entitled, while on service, to maternity and sanatorium benefits, and that they are eligible to return to full normal benefits without penalty on discharge. For these modified benefits soldiers have deducted out of their own pay a reduced contribution of 1½d. per week, whereas the normal contribution is 4d. We think that with these particulars before him our correspondent will agree that our Note was perfectly correct, both as to the facts and the inferences.

### THE PROPER FUNCTION OF A SANATORIUM.

As illustrating the ubiquity of a difficulty which is encountered by all tuberculosis officers and Insurance Committees a discussion at a recent meeting of the Shipley and Bingley District Insurance Committee deserves a passing notice. The difficulty arises from the fact that many patients fail to derive as much benefit as they should do from sanatorium treatment owing to their propensity to return home too early. The reasons for this attitude are various; in fact, almost every imaginable excuse has been put forward, except, of course, the one of expense of board and lodging at the sanatorium. It is to be feared that much too often the reason given for wishing to be discharged is an inadequate one; an optimistic notion that by "taking great care" of themselves when they return home the benefit of sanatorium life will go on working in their favour indefinitely is at the bottom of many of such requests. Naturally it must happen that after a prolonged stay in an institution the progress of a patient comes to a standstill by reason of the monotony causing a depression which militates against the health-giving factors of the treatment, but the cases which are most insistent for discharge are those whose sojourn has been only a matter of two or three months at the most. Patients whose symptoms are of comparatively recent origin at their first visit are very hardly made to realise the importance of a prolonged stay in a sanatorium. When their disease has obtained a firmer foothold they are ready enough to clamour for readmission and not so ready to go; though at the latter period it is only possible to patch them up for six or twelve months. Patching is not the proper function of a sanatorium. Unfortunately, far too much patching has at present to be attempted in these institutions.

### THE WINTER SESSION.

The ceremonies which in normal times mark the opening of the winter session at the medical schools were dispensed with this year in all but a few cases, and at most of the schools the students begun their work on October 1 without formality. This is as it should be, for the present is the time for action, not for speech-making and social functions. With only one exception does the new session commence without depleted schools and reduced staffs, this exception being the London School of Medicine for Women, where the number of entrants is the largest on record. Here the opening address was delivered by Dr. Florence Willey, who pointed out that while the prevalent lack of labour had opened the doors of various industrial pursuits to women, in no calling was the demand for women more urgent than in the medical profession. But, unfortunately, the increase in the number of women students, however gratifying it may be, can only go a very little way towards filling gaps in the professional ranks. At the medical school of Leeds University, the new session was opened by Sir William Osler, who delivered an eloquent and characteristic address. At the Middlesex there was no formal opening address, but Sir James Kingston Fowler, one of the consulting physicians, said a few appropriate words to the assembled students. At Charing Cross Hospital Medical School the prizes were distributed by Dr. Ronald M. Burrows, Principal of King's College, who pointed to the record of the Universities, whose Officers' Training Corps had given to the New Army probably 80 or 90 per cent. of its officers, while the colleges and hospitals of London had done yeoman service in putting their students and their apparatus entirely at the service of the Government and the nation during the war. Truly a proud record!

### THE CURATIVE VALUE OF GUNPOWDER.

We are all just now only too vividly aware of the destructive and damaging qualities of gunpowder and hence to hear a proclamation of its curative virtues is an experience which creates surprise. Yet it is announced, apparently in all seriousness, that gunpowder—it is the old-fashioned black variety which is intended—has considerable therapeutic merits. Specially appropriate to the hour is the intimation that it is a highly successful application to wounds produced by firearms. Thus it would seem to conform to a doctrine familiar to a certain group of persons whose constitutions need a minimum scheme of dosage, as also to others who are accustomed to look upon the wine when it is red. Pushing the proposal a further stage, gunpowder is alleged to contain ingredients which "purify the blood," and this without the endorsement of a Government stamp. From wounds it is therefore an easy step to the position that many other of the ills to which flesh is heir, such as boils and skin diseases, yield to the black agent, while clearly the poisons introduced by the bites of serpents, snakes, scorpions, insects, and other creeping things recognise that here at last must their proud triumphs be stayed. Such, briefly, are the claims. The witnesses to character follow in due course. First come our old friends the Indians (colour not stated, but presumably the red variety) of North America, who are accustomed to "see snakes" in the most literal sense of the term, and from whose tribal pharmacopœia have been obtained so many of the delightful remedies which excite the adjectives of our modern advertisement agents. Next follows that picturesque figure, "the soldier of the old days," who has ever been known as a fire-eater, and may well, therefore, as we are told, have made a habit of swallowing gunpowder in teaspoonful doses mixed with hot water; proverbially the soldier is "full of strange oaths," and if his habits are here correctly defined the vigour of his vocabulary is hardly a cause for wonder.

### GUNPOWDER, SHEPHERDS, AND PARSONS.

To pass to more peaceful realms, we find next that "shepherds in East Anglia"—the wisdom of the East is notorious—add gunpowder to their bread and cheese, and regard it as a "dressing" which has both "curative and remedial" properties. Not only for their own persons but also for their flocks are these properties invoked, and, learning this, we may hesitate to think what may be said of these good shepherds in quarters where the quadruped stirs the deepest emotions. Last, and as a final illumination, comes the testimony of a pastor in a still higher sphere—none other than a "sensible rector," who, braving all the displeasure of his ecclesiastical superiors, administers the explosive remedy wherever his official cure of souls extends, and this, it is said, to the great advantage of the parish. Mrs. Squeers enforced scholastic discipline by the aid of brimstone, and she has had not a few theological imitators. From brimstone to gunpowder is not a long cry, and it is easy to imagine how steadily all eyes will be directed towards the pulpit, and how fervid will be the responses, when the congregation is aware that lukewarmness or slumber may entail not only tortures in the remote future, but also gunpowder in the day which now is. The "sensible rector" may have many claims to attention; it is right that here we should particularly note his skill in promoting in a single successful stroke the attainment both of therapeutic achievement and of ecclesiastical discipline. Taking the testimonies all round, it is pleasant to reflect that at a time when gunpowder appears as an agent of suffering and death it has friends who would fain rehabilitate it. We are taught that there are "sermons in stones"; in similar fashion it seems there is good in gunpowder.

### THE MANCHESTER ELECTION.

A great deal of interest has necessarily centred round the appointment of a new superintendent and secretary of the Manchester Royal Infirmary. Every candidate must have held office as a general superintendent or secretary of a voluntary hospital. There were thirty-six candidates, of whom eleven did not comply with the terms of the advertisement and were disqualified. The remaining twenty-five candidates were men of varying experience in hospital work, and it was not easy to reduce them to seven, to constitute the first list of candidates to appear before the Selection Committee. The Selection Committee took infinite pains and had no easy task in choosing three of the seven to submit to the Board of Management, with whom the responsibility of the appointment rested. Of the three one was the secretary-superintendent of a large general hospital in the North, another the secretary and general superintendent of an excellently administered hospital in Wales, and the third was Mr. Frank G. Hazell, secretary of the Norfolk and Norwich Hospital, Norwich. In the result the Board of Management appointed Mr. Hazell at a commencing salary of £700 per annum, with an unfurnished flat in the infirmary, washing, fuel, and light. Mr. Hazell's application was a model of conciseness, modesty, and convincing force. It could not fail to carry great weight with a business committee, and the testimonials given by the Chairmen of the Board of Management, of the House Committee, Finance, Hon. Medical Staff, and Nursing Committees united in telling commendation of the high and exceptional qualifications of the successful candidate.

### MR. FRANK G. HAZELL.

From the age of sixteen upwards Mr. Hazell has had continuous experience in hospital administration, having been for nine years an assistant to the secretary at St. Luke's Hospital, London, and for fifteen years secretary and chief executive officer at the Norfolk and Norwich Hospital. Mr. Hazell has also been responsible for the administration of the Fletcher Convalescent Home, Cromer, and has had wide experience in connection with the staff of private nurses at Norwich. Since the commencement of the war he has had much extra work (in which he has been assisted by two voluntary workers) in connection with the addition of 250 beds at the Norwich Hospital for soldiers, the number admitted having been approximately 3,000. During his tenancy of office the ordinary income of the Norwich Hospital has increased from £8,450 to £13,830. As an administrator Mr. Hazell has proved a remarkable success. This may be gathered by reference to the report of an inspection of this hospital which appeared in THE HOSPITAL of January 3, 1914, page 369 *et seq.* This report may be read with encouragement by all the younger hospital men throughout the country. Mr. Hazell's record is excellent. He is a gentlemanly, capable man, who impresses a visitor, especially one who has a thorough knowledge of hospital administration. In congratulating Mr. Hazell upon his appointment we may properly add out of fulness of knowledge that his twenty-four years' previous work for voluntary hospitals and its success mark him out as the right man in the right place. The administration of the Norfolk and Norwich Hospital under Mr. Hazell has become so efficient as to make this hospital an example of good management amongst all the voluntary hospitals of East Anglia.

### MOTHERS' RIGHTS AND GUARDIANS' WRONGS.

FOR some time the Lambeth Board of Guardians have been urged to grant more facilities for mothers in their workhouses at North Lambeth to visit their young babies, which have been removed to a nursery at Norwood, some miles away. One visit a month is all that is allowed at the present time, and an effort has been made to increase these days of visitation to once a week, but in vain. The Guardians stand by their rule. It is argued that the rule as at present existing tends to destroy the maternal instinct, and facts are adduced to show that when children have been in these institutions for years, with few visits from their mothers, they lose all sense of love for their parent. When the children were in the workhouses the mothers were able to see them daily, so that they could maintain their interest in their offspring. Now the widening gulf has to a great extent helped to kill this feeling of motherhood, and in years to come the breach between the mother and her child may become complete. The conditions as laid down by the Lambeth Board of Guardians savour of Bumbledom, for the expense of letting the mothers more frequently visit their babies is trifling. The Guardians must realise that they have a much wider responsibility towards these women and children than that of placing the latter in nurseries. Indeed, the mutual right of mother and child to frequent meetings is one which cannot be sacrificed to red tape; for public institutions of this kind must come to the rescue of the home. Attempts to destroy it, like the Lambeth example, if persisted in, can only lead to the supersession of the authorities responsible. We refer to another aspect of the question on page 23.

### THE TRADE DEFENCE OF ADULTERATION.

DR. R. KAYE BROWN, medical officer of health for the Borough of Bermondsey, has presented to his Public Health Committee a lengthy report dealing with the dangers arising to public health through using in the manufacture of food raw materials that are either unsound or partially unsound. Owing to trade competition and a general demand for cheap and showy articles of food regardless of their physiological food value, there was, he regretted to say, a tendency amongst some importers of and wholesale dealers in food, manufacturers of the cheaper classes of food, and public caterers to adopt a lower standard and to use any sort of raw materials provided the final product were made to appear wholesome by the addition of aromatic substances, or by the process of cooking. If taxed with using such materials they replied that better-class ones cost more, that the articles in question were not injurious to health, and that the public were benefiting by the reduction in price. Many attempts had been made to introduce unsound raw materials in the manufacture of food, and no doubt more attempts would be made but for the vigilance of the sanitary authorities. Unfortunately, these authorities did not always succeed, for the pecuniary interests involved were so great that interested parties did not hesitate to go to great expense and trouble in getting expert evidence in their favour, but with the best intentions in the world trade experts and analytical chemists had done their work when they had informed the Court as far as they could whether the article of food analysed by them consisted of its normal constituents, and if abnormal ones were present, their nature and amount. He looked upon it as a presumption to apply the coarser methods of the ordinary chemical laboratory to the highly complicated physiological processes of the human body, and to endeavour from them to draw conclusions of the possible effects of this or that drug or article of food under the varying conditions of health and disease.

### THIS WEEK'S DRUG MARKET.

ON the whole business in the drug market has been somewhat quieter during the past week, but in some departments there has been increased activity. For instance, makers of bismuth preparations and mercurials continue so busy that they cannot execute orders promptly, and the same applies to some of the other products for which an abnormal demand has been created by the war; owing to the very heavy orders, corrosive sublimate has been advanced in price. On the other hand, the continued advance in the prices of synthetic drugs has the effect of restricting the demand to very narrow limits; extraordinary high figures are now asked for some of these, and several of them are obtainable only with difficulty. To the persistent advance in the price of phenacetin there seems to be no end, while salicylic acid, sodium salicylate, and acetyl-salicylic acid again show a tendency to move upwards. Cod-liver oil is without material change, but with the chief consuming season so close at hand buyers will be compelled to come on to the market before long, and there does not appear to be any great prospect of a substantial decline in value. American peppermint oil continues to tend upwards in price, and quotations for the English oil are higher. Senega is somewhat easier—as a result of supplies being more plentiful. Ipecacuanha is dearer. Extreme prices are being paid for caffeine. Epsom salt is tending downwards in price.

